# Hierarchical Knowledge Transfer: Cross-Layer Distillation for Industrial Anomaly Detection

**DOI:** 10.3390/jimaging11040102

**Published:** 2025-03-28

**Authors:** Junning Xu, Sanxin Jiang

**Affiliations:** College of Electronics and Information Engineering, Shanghai University of Electric Power, Shanghai 201306, China; xujunning@mail.shiep.edu.cn

**Keywords:** anomaly detection, knowledge distillation, cross-layer transfer, attention mechanism, feature extraction

## Abstract

There are two problems with traditional knowledge distillation methods in industrial anomaly detection: first, traditional methods mostly use feature alignment between the same layers. The second is that similar or even identical structures are usually used to build teacher-student models, thus limiting the ability to represent anomalies in multiple ways. To address these issues, this work proposes a Hierarchical Knowledge Transfer (HKT) framework for detecting industrial surface anomalies. First, HKT utilizes the deep knowledge of the highest feature layer in the teacher’s network to guide student learning at every level, thus enabling cross-layer interactions. Multiple projectors are built inside the model to facilitate the teacher in transferring knowledge to each layer of the student. Second, the teacher-student structural symmetry is decoupled by embedding Convolutional Block Attention Modules (CBAM) in the student network. Finally, based on HKT, a more powerful anomaly detection model, HKT+, is developed. By adding two additional convolutional layers to the teacher and student networks of HKT, HKT+ achieves enhanced detection capabilities at the cost of a relatively small increase in model parameters. Experiments on the MVTec AD and BeanTech AD(BTAD) datasets show that HKT+ achieves state-of-the-art performance with average area under the receiver operating characteristic curve (AUROC) scores of 98.69% and 94.58%, respectively, which outperforms most current state-of-the-art methods.

## 1. Introduction

In modern industrial manufacturing, quickly and accurately detecting and locating anomalies is crucial for ensuring product quality. With the successful application of deep learning models, particularly Convolutional Neural Networks (CNNs), in various computer vision fields, many deep learning-based anomaly detection methods [1] for industrial scenarios have emerged. These methods have shown great potential in various practical applications, such as large-scale industrial manufacturing, video surveillance, and medical diagnosis. However, in real-world industrial scenarios, the scarcity of defect samples, high annotation costs, and lack of prior knowledge about defects can render supervised methods ineffective. Therefore, most of the current research focuses on unsupervised anomaly detection methods [2,3,4,5]. This means that the training set only contains normal samples, while the test set includes both normal and anomalous samples. This makes constructing fast and accurate anomaly detection methods highly challenging.

In recent years, unsupervised approaches based on knowledge distillation [6,7] have garnered widespread attention due to their outstanding performance and lower resource consumption. These methods [6,8,9] employ student-teacher (S-T) models to transfer meaningful knowledge from other computer vision tasks pre-trained on natural datasets. Specifically, a partial layer of a backbone network pre-trained on a large-scale dataset is usually selected as the fixed-parameter teacher model. During training, the teacher model imparts the knowledge of extracting normal sample features to the student model. Conversely, during inference, the features extracted from normal samples by the teacher and student networks are similar, while the features extracted from abnormal samples are quite distinct. By comparing the differences in the feature maps generated by the two networks, it is possible to determine whether the test image is abnormal and locate the anomaly. Bergmann et al. [10] pioneered the introduction of the T-S architecture to abnormality detection, which significantly outperforms other benchmark methods. However, the model only uses the output of the last layer of the teacher network as a feature for knowledge distillation. To enhance knowledge transfer, STPM [11] utilizes multi-scale features distilled under different network layers. In this case, for normal samples, the features extracted from the student network are more similar to those extracted from the teacher network. In contrast, for abnormal samples, the extracted features differ more. However, these methods are prone to produce redundant features at multiple scales. To solve this problem, RD4AD [12,13] further proposes the Multi-scale Feature Fusion (MFF) module and One-Class Bottleneck (OCB) to form embeddings, which enable a single pair of T-S networks to reconstruct features efficiently. However, the student network in RD4AD needs to receive inputs from the instructor network, which makes it easy for the instructor network to leak anomaly information to the student network during testing.

**Figure 1:**
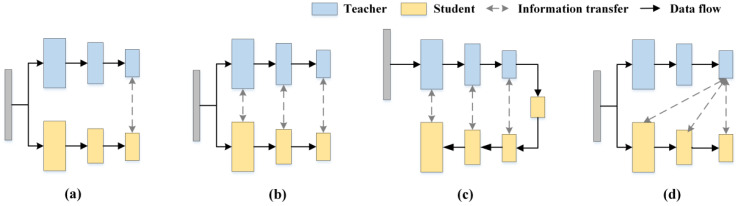
Knowledge transfer methods in knowledge distillation. (**a**) Deepest-layer knowledge transfer. (**b**) Multi-scale transfer architecture. (**c**) Reverse distillation paradigm. (**d**) HKT/HKT+.

The study found that although these knowledge distillation-based anomaly detection methods show potential in industrial scenarios, these methods usually adopt similar or even identical structures for constructing S-T models, thus limiting the ability of S-T networks to represent anomalies in diverse ways. In addition, most of these approaches use the same inter-level feature alignment (e.g., Teacher Layer 4→Student Layer 4), which results in deep semantic knowledge in the teacher network not being efficiently transferred to the student network. For a clearer description, knowledge distillation-based anomaly detection methods can be briefly categorized by knowledge transfer, as shown in Figure 1. Figure 1a represents an approach to knowledge transfer using only the deepest levels of the teacher and student [14]; Figure 1b represents the T-S network for knowledge transfer through multi-scale [11]; Figure 1c represents the approach of reverse distillation paradigm [12,13]; While HKT/HKT+ utilizes the deepest features of the teacher network to supervise the learning at each stage of the student network, which is distinctly different from previous knowledge transfer methods. This is shown in Figure 1d.

**Figure 2:**
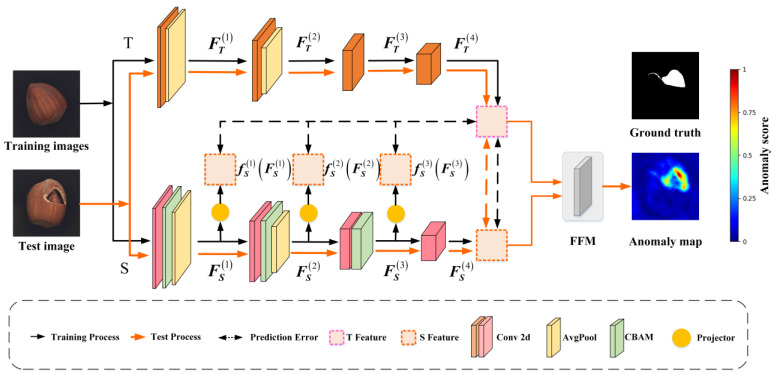
The architecture of HKT and the knowledge transfer mechanism between S and T.

Currently, exploring efficient knowledge transfer techniques tailored for anomaly detection remains a research priority. To address the above-mentioned issues, the following improvements are introduced:

- A knowledge transfer framework for anomaly detection, termed HKT, is proposed, as illustrated in Figure 2. This method utilizes the deep features extracted from the teacher network to guide the feature extraction at each stage of the student network, which is significantly different from the traditional knowledge transfer methods that operate only between corresponding layers. This cross-layer transfer method can effectively improve the imitation ability of the student network.
- A lightweight yet effective attentional mechanism, the CBAM [15], is introduced between the student’s convolutional layers. CBAM enhances both the channel and spatial dimensions and strengthens anomaly-sensitive regions by cascading them. These further increase the difference in anomaly representations between the teacher network and the student network, thus improving the anomaly detection accuracy of HKT. In addition, the CBAM module is lightweight and suitable for real-time tasks.
- A more robust anomaly detection model, HKT+, is developed on top of HKT. By adding two convolutional layers to the teacher and student networks of HKT, HKT+ achieves stronger detection at the cost of relatively small model parameters and increased computational complexity.
- Extensive experiments and analyses on the anomaly detection datasets MVTec AD [16,17] and BTAD [18] demonstrate that both HKT and HKT+, as lightweight models, outperform most state-of-the-art anomaly detection methods.

## 2. Materials and Methods

### 2.1. Framework

The overall architecture of HKT is illustrated in Figure 2. It consists of a Teacher network (T), a Student network (S), and a Feature Fusion Module (FFM). Specifically, the Teacher network T has been pre-trained on an image classification dataset. In contrast, the Student network S is trained solely on normal images under the guidance of the Teacher. The Teacher network T adopts a CNN architecture, extracting deep features from input images through a four-stage convolutional process, and guides the Student network S via projectors. The Student network progressively extracts features from input images through its four-stage convolutional process. Since the structure of S is similar to that of T, the features extracted by the student and the teacher should be highly correlated when S tries to fit the output of T accurately.

However, the shallow features of S are not in the same space as the deep features extracted by the teacher. Therefore, three projectors are designed in the student part to map their features into the teacher’s space. With the assistance of these projectors, knowledge is efficiently transferred from T to S. Finally, the FFM integrates feature maps from both networks to generate pixel-wise anomaly scores for anomaly detection.

### 2.2. T-S Network

The teacher network employs the Patch Description Network (PDN) [14], which contains only four convolutional layers. This significantly reduces network depth while maintaining its effectiveness as a feature extractor. The PDN is fully convolutional and can be applied to images of varying sizes, generating all feature vectors in a single forward pass. By incorporating average pooling layers after the first and second convolutional layers, the PDN substantially reduces computational costs.

**Figure 3:**
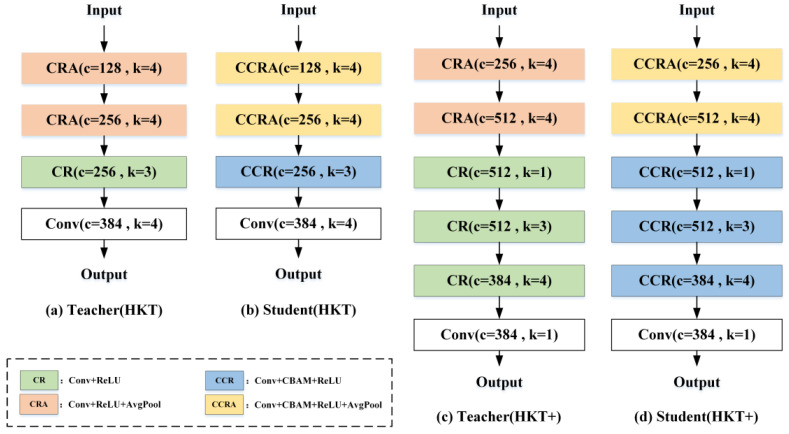
Detailed structure of the HKT/HKT+ network.

The student network S shares a similar architecture to the teacher network T but introduces attention mechanisms in its first three stages. Additionally, to ensure feature dimensions at each stage of the student network match those of the teacher network, an interpolation function is integrated as a projector in each stage. Furthermore, for CNN architectures, deeper networks with more convolutional layers generally exhibit stronger feature extraction capabilities. To leverage this, both the teacher network T and student network S in HKT are enhanced with two additional convolutional layers, resulting in a more powerful anomaly detection network named HKT+. The specific architectures of the teacher and student networks in HKT and HKT+ are shown in Figure 3. Here, (a) denotes the teacher structure of HKT; (b) denotes the student structure of HKT; (c) denotes the teacher structure of HKT+; and (d) denotes the student structure of HKT+. Based on HKT, the number of kernels in the hidden convolutional layers of HKT+ teachers and students is doubled. HKT+ also inserts a 1 × 1 convolution after the second pooling layer and the last convolutional layer in HKT. The main difference between HKT/HKT+ teachers and students is whether CBAM is added or not.

### 2.3. Attention Mechanism

The introduction of the attention mechanism [19,20] helps student network S to focus on the guidance provided by teacher network T from its initial stages, thereby mimicking the teacher’s final output. However, in the field of anomaly detection, traditional attention mechanisms are often limited by unidimensionality or computational redundancy. CBAM, with its two-dimensional attention synergy, computational efficiency, and anomaly sensitivity optimization capability, is ideal for the structure of the student network in this study. In particular, the channel attention module of CBAM learns the importance of weights on the channel dimension to enhance the response of critical channels. The spatial attention module learns importance weights on the spatial dimension to focus on critical regions. Moreover, the small number of parameters of CBAM has the advantage of being lightweight and suitable for real-time tasks.

**Figure 4:**
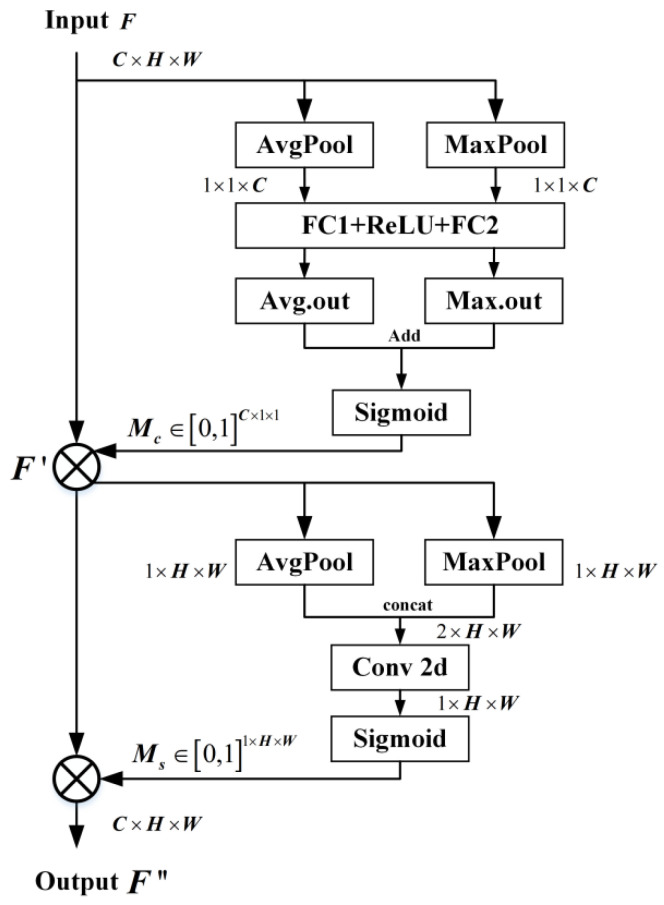
The flowchart of the CBAM module.

The flowchart of the CBAM structure is shown in Figure 4. The output of the channel attention module is formulated as follows:

(1) $$M_{c}\left(F\right)=\sigma \left(W_{2}\left(\delta W_{1}\left(\mathrm{AvgPool}\left(F\right)\right)\right)+W_{2}\left(\delta W_{1}\left(\mathrm{MaxPool}\left(F\right)\right)\right)\right)$$

where F is the input feature map; AvgPool and MaxPool denote the full-drama average pooling and maximum pooling operations, respectively; $W_{1}$ and $W_{2}$ represent the two fully connected layers, respectively; δ represents the ReLU activation function, and σ denotes the sigmoid activation function.

The output of the spatial attention module is formulated as follows:

(2) $$M_{s}\left(F^{\prime }\right)=\sigma \left(f^{7\times 7}\left(\left[\mathrm{AvgPool}\left(F^{\prime }\right);\mathrm{MaxPool}\left(F^{\prime }\right)\right]\right)\right)$$

where $f^{7\times 7}$ denotes a 7 × 7 convolution operation and $\left[\mathrm{AvgPool}\left(F^{\prime }\right);\mathrm{MaxPool}\left(F^{\prime }\right)\right]$ denotes the stitching of the average pooling and maximum pooling results along the channel axis.

(3) $$\begin{array}{l} F^{\prime }=M_{c}\left(F\right)\times F\\ F^{\prime \prime }=M_{s}\left(F^{\prime }\right)\times F^{\prime } \end{array}$$

CBAM is applied after the convolutional layers of the first three stages (for simplicity, the last stage of S does not include the attention mechanism). With channel attention $M_{c}$ and spatial attention $M_{s}$, the feature map output is $F^{\prime \prime }$. CBAM can effectively focus on important features while suppressing less useful features, thus enhancing the ability of the student network, S, to mimic the teacher’s network, T.

Additionally, the introduction of CBAM creates an obvious architectural difference between the student network S and the teacher network T, which enhances the distinctiveness of their anomaly representations. Notably, CBAM inherently focuses on the guidance provided by the teacher network T. By incorporating CBAM both solves the problem of identical T-S architectures and, at the same time, forces the student network to mimic the important regions emphasized by the teacher. This effectively improves the student network S’s ability to extract features from normal data and amplifies the disparity between features of anomalous and normal data extracted by S, thereby enhancing anomaly detection performance.

### 2.4. Projector and Loss

Since the student network S and the teacher network T share a similar 4-stage CNN architecture, their final outputs, $F_{S}^{\left(4\right)}$ and $F_{T}^{\left(4\right)}$, are in the same feature space and have the same dimensions. However, the features extracted by the student network at stages 1, 2, and 3 ($F_{S}^{\left(1\right)}$, $F_{S}^{\left(2\right)}$, and $F_{S}^{\left(3\right)}$) are not in the same feature space as $F_{T}^{\left(4\right)}$. To facilitate knowledge transfer, we constructed three projectors, $f_{S}^{\left(1\right)}$, $f_{S}^{\left(2\right)}$, and $f_{S}^{\left(3\right)}$, which use nearest neighbor interpolation to downsample the features, $F_{S}^{\left(1\right)}$, $F_{S}^{\left(1\right)}$, and $F_{S}^{\left(3\right)}$, respectively. This ensures that the resulting outputs, $f_{S}^{\left(1\right)}(F_{S}^{\left(1\right)})$, $f_{S}^{\left(2\right)}(F_{S}^{\left(2\right)})$, and $f_{S}^{\left(3\right)}(F_{S}^{\left(3\right)})$, match the dimensions of $F_{T}^{\left(4\right)}$.

HKT supervises the learning of a network of students through multi-stage losses, as defined in the following:

At stage 4, the student network is in the same feature dimension as the teacher network at the final output layer, with global semantic alignment. Therefore, the loss function of the student network S can be defined as follows:

(4) $$L^{\left(4\right)}=\left\|{\left.F_{T}^{\left(4\right)}-F_{S}^{\left(4\right)}\right\|}_{2}^{2}\right.$$

If the student input feature map for each layer is $F_{S}^{\left(i\right)}\in R^{C^{i}\times H^{i}\times W^{i}}$, the projection operation can be formalized as follows:

(5) $$f_{S}^{\left(i\right)}(F_{S}^{\left(i\right)})=\mathrm{Interpolate}(W_{1\times 1}*F_{S}^{\left(i\right)},\mathrm{size}=[H^{4},W^{4}])$$

where $W_{1\times 1}$ denotes the 1 × 1 convolutional kernel weights used to adjust the first three levels of the student’s channel dimensions to 384 dimensions (i.e., the teacher’s channel dimensions) and Interpolate(⋅) denotes a nearest neighbor interpolation operation that adjusts the spatial dimensions from to the target dimension (i.e., the teacher’s size).

Assuming the total loss of the student network S at stages 1, 2, and 3 is $L^{\left(P\right)}$, it is defined as follows:

(6) $$L^{\left(P\right)}=\sum_{i=1}^{3}\left\|{\left.F_{T}^{\left(4\right)}-f_{S}^{\left(i\right)}(F_{S}^{\left(i\right)})\right\|}_{2}^{2}\right.$$

Then, the total loss of this student is as follows:

(7) $$L_{total}=L^{\left(4\right)}+\lambda_{1}L^{\left(P\right)}$$

Here, $\lambda_{1}$ is a hyperparameter. $\lambda_{1}$ is usually set to 0.5.

### 2.5. Feature Fusion Module

**Figure 5:**
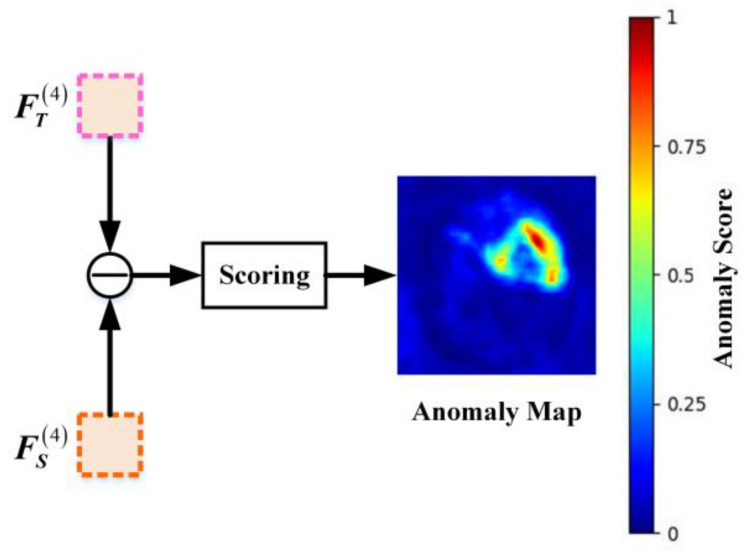
Detailed structure of the FFM module.

During the testing phase, the mean squared error (MSE) between corresponding elements of the Teacher’s and Student’s outputs is calculated to quantify their feature discrepancies, as defined in Equation (4). The architecture of the Feature Fusion Module (FFM) in HKT is illustrated in Figure 5.

The FFM integrates semantic discrepancies between the Teacher network T and Student network S. By aggregating these discrepancies; the FFM assigns an anomaly score to each pixel of the input image based on the magnitude of the differences, ultimately generating a pixel-wise anomaly map.

## 3. Experiments

### 3.1. Datasets

In this study, the following two commonly used public industrial anomaly detection datasets are selected to evaluate the HKT/HKT+ model.

- MVTec AD Dataset

This dataset is designed for benchmarking anomaly localization algorithms and includes a total of 5354 high-resolution images of industrial products, covering 15 different categories. Each category’s training set contains only defect-free images, while the test set includes both normal images and various types of anomaly images. Defect types include surface defects (such as scratches and dents), structural defects (such as deformation of object parts), and defects involving missing parts. The distribution of the MVTec AD samples is shown in Table 1.

- BTAD Dataset

This dataset specializes in object detection and semi-supervised learning tasks. It is commonly used in manufacturing and anomaly detection studies. The dataset includes RGB images of three different industrial products (Class 1, Class 2, and Class 3), totaling 2540 images. It includes 1799 images for training and 741 images for testing. The distribution of BTAD samples is shown in Table 2.

### 3.2. Implementation Details

All experiments in this study were conducted using an NVIDIA GeForce RTX 4090 GPU. Following the reference [14], the teacher network employs the PDN structure pre-trained with WideResNet-101 [5] on the ImageNet dataset [21], which has strong feature representation capabilities, and the detailed flowchart is shown in Figure 3a. The student network’s parameters were randomly initialized. To expedite training, the input high-resolution raw images were scaled to 256 × 256 × 3 pixels for both training and testing. The network was trained using the Adam optimizer [22], with an initial learning rate of 0.0001, a weight decay coefficient of 0.00001, a batch size of 1, and 50,000 training iterations.

### 3.3. Evaluation Criteria

For image-level anomaly detection, the Area Under the Receiver Operating Characteristic Curve AUROC [23], the number of parameters, and Floating-Point Operations (FLOPs) are used as evaluation metrics.

AUROC measures the relative values of the True Positive Rate (TPR) and the False Positive Rate (FPR) at different thresholds and evaluates the best potential segmentation results based on normal and abnormal pixels. AUROC comprehensively captures the overall performance of the model in the classification task and takes the value ranging from 0 to 1. Its physical significance can be interpreted as follows: if AUROC = 0.5 indicates that the model performance is equivalent to random guessing and cannot provide effective predictive value for practical applications if AUROC is greater than 0.9, the model is usually considered to have industrial-grade practical value. If AUROC tends to 1, it indicates that the model has perfect classification ability and can accurately perform classification. That is, the larger the AUROC value, the better the model performance. The number of parameters is used to assess the degree of model lightweight. One GFLOPs is equal to one billion (=10^9^) floating point operations per second, which is used to quantify the computational complexity of the model.

### 3.4. Experimental Results and Analysis

To evaluate the performance of the algorithms proposed in this paper, this study compares the performance of the algorithms with the current leading anomaly detection methods on two classical defect datasets, including DRAEM [24], STPM [11], RD4AD [12], MKD [25], US [10], FABLE [26], CSFlow [27], CFlow-AD [28] and SCL [29] Among these methods, DRAEM is based on reconstruction. CSFlow and CFlow-AD are flow-based methods, SCL adopts a self-supervised learning method, and the rest are based on feature knowledge distillation theory. We have also constructed two different sizes of feature-based T-S models; the former is lightweight, and the latter is medium-sized. Its derived anomaly detection methods are HKT and HKT+.

#### 3.4.1. Performance on Dataset MVTec AD

The detection accuracy of HKT and HKT+ was compared with classical anomaly detection methods on the MVTec AD dataset using AUROC as the evaluation metric. As shown in Table 3, HKT and HKT+ achieve average AUROC values of 97.34% and 98.69% across all 15 categories, respectively. These results significantly outperform other knowledge distillation-based methods, demonstrating the effectiveness of the distillation mechanism proposed in this study. Furthermore, HKT+ exhibits higher AUROC than HKT due to its deeper feature-based teacher-student network architecture.

For the five texture anomaly categories (Carpet, Grid, Leather, Tile, and Wood), HKT+ achieves AUROC values exceeding 98.5% for all classes, with no notable underperforming categories. Specifically, Leather attains a perfect 100% AUROC, while Grid and Tile approach near-perfect detection (99.8% and 99.7%, respectively). HKT achieves a competitive average AUROC of 98.99% for texture anomalies, whereas HKT+ further elevates this to 99.46%, surpassing all baseline methods.

Among the 10 object categories, HKT+ achieves AUROC values above 97% for all classes, with six categories exceeding 98% and four categories surpassing 99%. However, for Cable and Transistor, HKT+ yields relatively lower AUROC scores (~94%), underperforming compared to FABLE and US. This indicates potential areas for refinement in HKT+’s knowledge distillation framework, particularly for complex structural anomalies.

#### 3.4.2. Performance on Dataset BTAD

**Figure 6:**
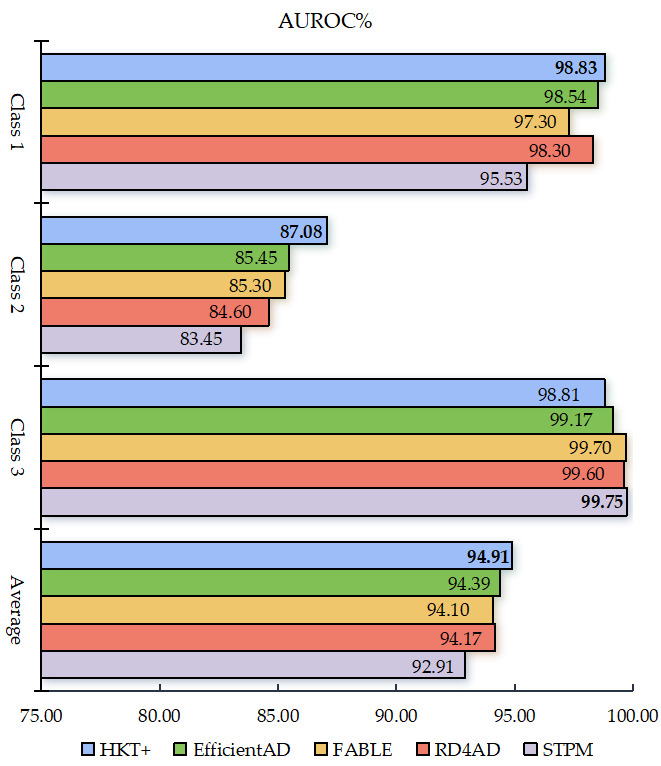
Comparison of anomaly detection performance based on the BTAD datasets.

To validate the generality and generalization ability of the model, the performance of HKT+ is further evaluated on the BTAD dataset. HKT+ is compared with four state-of-the-art methods: STPM [11], RD4AD [12], EfficientAD [14], and FABLE [26]. The results are illustrated in Figure 6. At least two key observations can be drawn: First, for anomalies across all three categories, HKT+ achieved an average AUROC exceeding 94.5%, outperforming all other methods whose average AUROC remained below this threshold. Notably, EfficientAD ranked second with an AUROC of 94.39%, demonstrating the superior performance of HKT+ on this dataset. Second, for the more challenging Category 2 anomalies, HKT+ exhibited exceptional robustness, achieving an AUROC above 87%, whereas the highest AUROC among other methods was only 85.45%.

#### 3.4.3. Visualized Results

**Figure 7:**
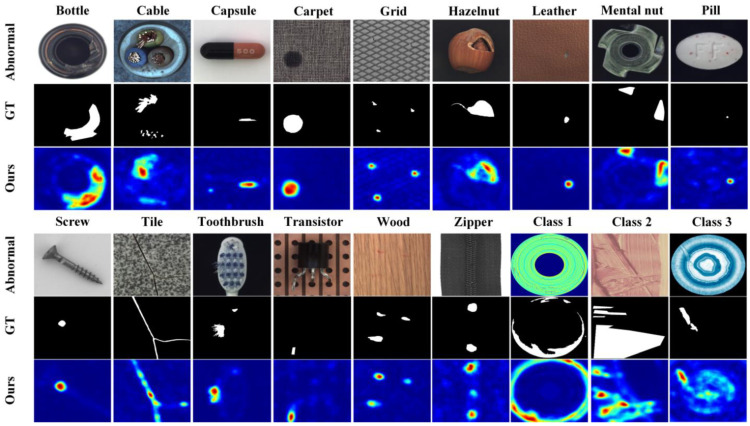
Typical anomalous images from the MVTec AD dataset and BTAD dataset, along with their corresponding ground truth and the detection results from HKT.

Figure 7 shows the typical anomaly samples, corresponding ground truth, and HKT detection results for the 18 categories included in the MVTech AD and BTAD datasets.

This figure shows that HKT accurately detects most of the anomalies in the test samples. However, a few anomalies may be missed. For example, in the Toothbrush sample, only one of the two defects was detected. There may also be false positives, such as in the Zipper and Class 3 samples, where normal parts are mistakenly identified as anomalies. Additionally, there can be inaccurate localization, such as in Class 2, where the anomaly was not precisely located. All these observations indicate that while HKT has strong anomaly detection capabilities, there is still room for improvement.

### 3.5. Complexity

The computational complexity (GFLOPs), number of parameters, and AUROC performance of HKT and HKT+ are compared with three state-of-the-art anomaly detection methods (DRAEM, US, RD4AD) on the MVTec AD dataset. The results are summarized in Table 4.

In terms of model size, HKT and HKT+ exhibit the smallest parameter counts, with approximately 3 M and 8 M parameters, respectively. This compact architecture enables seamless deployment in storage-constrained environments, significantly enhancing the method’s applicability and flexibility. Regarding computational efficiency, HKT and HKT+ require only 20 GFLOPs and 63 GFLOPs, respectively, substantially lower than most comparative methods. Such efficiency allows them to operate with reduced computational time and energy consumption in resource-limited scenarios. Most critically, HKT and HKT+ achieve impressive AUROC performance (97.34% and 98.69% on average), demonstrating competitive detection accuracy. By balancing lightweight design and high performance—two traditionally conflicting objectives—HKT provides a novel and promising solution for industrial anomaly detection with significant potential for real-world applications.

### 3.6. Ablation Study

For CNN architectures, deep networks with more convolutional layers typically exhibit stronger feature extraction capabilities. Consequently, HKT+ increases both the number of convolutional layers and the number of kernels in the hidden convolutional layers for both the teacher and student networks built on HKT. The depth extension variables of the HKT+ network were initially tested, and the results are presented in Table 5. If the depth had been increased without adding any other modules, the AUROC of HKT+ rises from 96.71% to 97.43% compared to HKT. However, the number of parameters (2.69 M → 8.02 M) and the computational cost (15.31 → 52.03 GFLOPs) of HKT+ increase significantly. This indicates that merely increasing the depth of the network can also substantially enhance performance, but it results in a greater computational burden.

In HKT/HKT+, the student network S has a similar architecture to the teacher network T, with the difference being the additional attention module CBAM. Moreover, to enhance the simulation capability of S, the projection loss $L^{\left(P\right)}$ is introduced between it and the teacher network. To validate the effectiveness of these two components, tests were conducted using HKT+ as an example, and the results are shown in Table 6.

As can be seen from the table, the performance of HKT is significantly lower when neither component is used, or only one of them is used compared to using both components at the same time. This indicates that Attention Mechanism and Projection Loss enhance the student’s performance from two different perspectives, and their effects are additive. HKT+ increases the number of parameters by 0.06 M (8.02 → 8.08) and GFLOPs by 0.04 (52.03 → 52.07) when only CBAM is added. However, AUROC increased by 0.8% (97.43% → 98.23%). This indicates that CBAM effectively focuses on key feature regions through the channel-space attention mechanism, suppresses extraneous noise, and significantly enhances the sensitivity of the model to local anomalies. It significantly improves the model performance at a very low computational cost. Without adding CBAM and only adding cross-layer loss $L^{\left(P\right)}$, the AUROC of HKT+ is improved to 97.71%, and the number of parameters and GFLOP are increased to 8.47 M and 63.18, respectively. This loss promotes cross-layer semantic fusion, solves the problem of severing the shallow texture information from the high-level semantic information, and optimizes the ability to locate complex defects but at a certain computational cost.

## 4. Discussion

### 4.1. HKT+ Performance Analysis

Analyzing the comprehensive performance, HKT+ significantly outperforms other mainstream methods in terms of AUROC and lightness on the MVTec AD dataset. The near or perfect detection in texture category tasks such as Carpet, Leather, and Hazelnut indicates that the model is extremely sensitive to the anomalies of uniform texture and regular structure. The excellent performance in object categories such as Bottle and Metal nut verifies the effectiveness of cross-layer knowledge migration $L^{\left(P\right)}$ for global semantic fusion. In addition, the HKT+ model accounts for lightweight design. Not only is the number of parameters and computational cost significantly lower than comparative methods such as RD4AD, but AUROC leadership is achieved at the same time. In terms of module design, the CBAM attention mechanism is added to the student module, which breaks the disadvantage of teacher-student isomorphism structurally and suppresses noise interference functionally by focusing on critical regions through channel space attention. Layered interactions in the teacher-student network are facilitated by cross-layer knowledge transfer loss $L^{\left(P\right)}$, which enhances the ability to localize complex defects. In texture-object mixing tasks (e.g., Tile, Zipper), the AUROC of HKT+ exceeds 99% in all cases. It is shown that the model effectively balances local details and global semantics for various defect types in industrial scenarios.

The low performance of some categories is due to the complexity of the structure and the smallness of the defects.

For example, the defect patterns of the Cable are mostly long structures (e.g., insulation breakage, core breakage); the Transistor has complex internal structures with tiny defects such as broken pins and package defects. It shows that the lightweight design of HKT+ may have limitations in detecting complex structures and tiny defects. Therefore, the extraction of deep semantic features by the HKT+ model needs to be optimized. In addition, visual analysis revealed that HKT+ also suffers from detection errors. For example, Class 2 and Zipper normal regions are misclassified as defects, which may be due to the over-sensitivity of spatial attention caused by the high texture background, which cannot effectively suppress the response of normal regions. Some defects on the Toothbrush are overlooked due to subtle breaks or deformations in the toothbrush bristles, which require finer pixel-level analysis.

To address these issues, we will continue to improve HKT+ in the future. Specifically, first, the attention mechanism will be improved by combining channel attention and pixel-level attention to enhance the localization accuracy of complex structural defects. Second, the generation and discrimination process of anomalous features will be optimized by drawing on the normalized flow design of CSFlow or the reconstruction design of DRAEM.

### 4.2. Balance Between Computing Resources and Performance

HKT prioritizes lightweight, low resource consumption, and ease of deployment. HKT+ trades computational costs for higher detection accuracy. But even so, HKT+ is lighter than most anomaly detection methods. HKT+ is better suited for scenarios that require high detection accuracy, such as cloud analytics, semiconductor wafers, and quality inspection of high-value products.

In real-world deployments, comprehensive decisions need to be made based on hardware conditions, real-time requirements, complexity of defect types, and data size. Example. For hybrid scenarios (e.g., one part of the production line requires real-time inspection, and another part requires high-precision review), a layered strategy can be used: HKT for initial screening and HKT+ for reviewing critical aspects to balance efficiency and accuracy.

## 5. Conclusions

Aiming at the problems of similar structure of teacher-student models and monotonicity of knowledge migration in traditional knowledge distillation anomaly detection networks, this paper proposes a cross-layer knowledge transfer method, HKT, for industrial anomaly detection. In HKT, the teacher guides the student network through multiple projectors. Utilizing the deep features in the teacher’s network to guide the features of the students in each layer can effectively improve the overall performance. Introducing the attention mechanism CBAM between convolutional layers can enhance the fusion of spatial semantic information. Experiments on the industrial surface defect datasets MVTec AD and BTAD datasets show that the proposed method achieves AUROC values of 98.69% and 94.58%, respectively. HKT is lightweight and suitable for deployment in resource-constrained scenarios such as edge devices and embedded systems. HKT+ has a higher deployment cost than HKT, but its detection accuracy exceeds that of most anomaly detection methods. It is suitable for applications that require very high detection accuracy.

HKT is an important benchmark for future anomaly detection research. In practice, HKT+ will be applied to devices such as high-performance embedded systems and FPGAs.

## Figures and Tables

**Table 1:** Sample distributions for fifteen categories in MVTec AD.

Category	#Train	#Test (Good)	#Test (Defective)	Image Side Length
Carpet	280	28	89	1024
Grid	264	21	57	1024
Leather	245	32	92	1024
Tile	230	33	84	840
Wood	247	19	60	1024
Bottle	209	20	63	900
Cable	224	58	92	1024
Capsule	219	23	109	1000
Hazelnut	391	40	70	1024
Metal Nut	220	22	93	700
Pill	267	26	141	800
Screw	320	41	119	1024
Toothbrush	60	12	30	1024
Transistor	213	60	40	1024
Zipper	240	32	119	1024
Total	3629	467	1258	-

**Table 2:** Sample distributions for the three categories in the BTAD dataset.

Category	#Train	#Test (Good)	#Test (Defective)	Image Side Length
01	400	21	49	1600 × 1600
02	399	30	200	600 × 600
03	1000	400	41	800 × 600
Total	1799	451	290	-

**Table 3:** Comparison of detection performance on the MVTec AD dataset using AUROC as the metric.

Category (Texture/Object)	Anomaly Detection Methods (AUROC%)										
	DRAEM [24]	STPM [11]	MKD [25]	RD4AD [12]	US [10]	FABLE [26]	CSFlow [27]	CFlow-AD [28]	SCL [29]	HKT (Ours)	HKT+ (Ours)
Carpet	97.0	98.9	79.3	98.9	99.9	99.0	**100.0**	99.3	96.4	97.68	98.88
Grid	99.9	**100.0**	78.0	99.3	98.5	91.6	97.6	99.0	97.0	99.92	99.92
Leather	**100.0**	99.9	95.1	99.4	**100.0**	**100.0**	97.5	99.7	99.2	**100.0**	**100.0**
Tile	99.6	95.5	91.6	95.6	99.0	**100.0**	98.8	98.0	99.5	99.93	99.82
Wood	99.1	99.2	94.3	95.3	97.9	99.4	**99.6**	96.7	99.5	97.46	98.68
Avg. Texture	99.1	98.7	87.7	97.7	99.1	98.0	98.7	98.5	98.32	98.99	**99.46**
Bottle	99.2	**100.0**	99.4	98.7	**100.0**	**100.0**	**100.0**	99.0	99.9	99.92	**100.0**
Cable	91.8	92.3	89.2	97.4	97.6	99.3	**100.0**	97.6	90.9	87.99	94.12
Capsule	98.5	88.0	80.5	98.7	85.3	84.6	**99.3**	99.0	86.7	97.61	98.48
Hazelnut	**100.0**	**100.0**	98.4	98.9	99.9	99.4	96.7	98.9	99.1	99.86	**100.0**
Metal nut	98.7	**100.0**	73.6	97.3	99.0	**100.0**	92.0	98.6	97.0	99.46	99.90
Pill	98.9	93.8	82.7	98.2	88.3	90.9	**99.1**	99.0	86.6	97.52	98.61
Screw	93.9	88.2	83.3	**99.6**	91.9	84.4	99.5	98.9	89.6	94.49	96.97
Toothbrush	**100.0**	87.8	92.2	99.1	95.0	86.7	95.2	98.9	99.9	98.61	97.50
Transistor	93.1	93.7	85.6	92.5	**100.0**	97.1	99.0	93.3	96.8	90.50	94.08
Zipper	**100.0**	93.6	93.2	98.2	96.7	95.3	98.7	99.1	99.1	99.16	99.58
Avg. Object	97.4	93.7	87.8	97.9	95.4	93.8	97.95	**98.2**	94.56	96.51	97.92
Avg. Total	98.0	95.5	87.7	97.8	96.6	95.2	98.2	98.3	95.81	97.34	**98.69**

Note: The best results are highlighted in bold.

**Table 4:** Comparison of HKT and HKT+ with other typical lightweight anomaly detection methods on the MVTec AD dataset.

Methods	Parameters (M)	GFLOPs	AUROC
DRAEM	97.4	198.4	97.98%
US	26.4	1948.1	96.60%
RD4AD	108.67	36.05	97.80%
HKT	**2.85**	**20.31**	97.34%
HKT+	8.52	63.21	**98.69%**

Note: The best results are highlighted in bold.

**Table 5:** Performance Comparison of HKT/HKT+ without CBAM and without Cross-Layer Knowledge Migration.

Methods	AUROC	Parameters (M)	GFLOPs
HKT	96.71%	**2.69**	**15.31**
HKT+	**97.43%**	8.02	52.03

Note: The best results are highlighted in bold.

**Table 6:** The effect of adding different modules on the detection accuracy of HKT+.

Modules		Metrics		
CBAM	$L^{\left(P\right)}$	AUROC	Parameters (M)	GFLOPs
×	×	97.43%	**8.02**	**52.03**
√	×	98.23%	8.08	52.07
×	√	97.71%	8.47	63.18
√	√	**98.69%**	8.52	63.21

Note: × means the module is not added; √ means the module is added. The best results are highlighted in bold.

## Data Availability

The datasets used in this paper are public datasets. The MVTec AD dataset is a public dataset that is openly available at https://www.mvtec.com/company/research/datasets/mvtec-ad/downloads (accessed on 26 March 2025). The BTAD dataset is also public and can be accessed at https://avires.dimi.uniud.it/papers/btad/btad.zip (accessed on 26 March 2025).
